# Variations in the *TNFα* gene and their interactions with the *IL4R* and *IL10* genes in relation to hand osteoarthritis

**DOI:** 10.1186/1471-2474-15-311

**Published:** 2014-09-24

**Authors:** Satu Hämäläinen, Svetlana Solovieva, Tapio Vehmas, Päivi Leino-Arjas, Ari Hirvonen

**Affiliations:** Finnish Institute of Occupational Health, Centre of Expertise for Health and Work Ability, Topeliuksenkatu 41 a A, FI-00250 Helsinki, Finland

**Keywords:** Tumor necrosis factor alpha, Gene polymorphism, Individual susceptibility, Hand osteoarthritis, Inflammation

## Abstract

**Background:**

The development of osteoarthritis (OA) involves inflammation, but the evidence for participation of genes propagating or inhibiting inflammation in the OA process is inconsistent. We investigated the associations of common variants in the *TNFα* gene, and their interactions with other cytokine genes, with hand OA among Finnish women.

**Methods:**

This cross-sectional study was based on bilateral hand radiographs of 542 female dentists and teachers which were classified according to the presence of OA (radiographic K-L score ≥ 2 in ≥ 3 joints) using reference images. The genotypes were determined by PCR-based methods. The degree of pairwise linkage disequilibrium (LD) and haplotypes were constructed and analyzed by the SNPStats software. The associations between four TNF*α* SNPs and hand OA were tested using logistic regression adjusting for age, occupation, and BMI, and fitting a log-additive model of inheritance. Gene-gene interactions of *TNFα* SNPs with *IL4R* and *IL10* SNPs were examined by stratified logistic regression analyses. Possible interactions of the *TNFα* SNPs with variants in the previously reported *IL1β* and *IL6* genes in influencing hand OA were also explored.

**Results:**

Two *TNFα* polymorphisms (*“-1031”* and *“-863*”) were associated with hand OA (OR = 1.45, 95% CI 1.01-2.07 and 1.55, 1.06-2.25, respectively). These associations retained when adjusting further for *IL1β* “3954” and *IL6* “174”. The *TNFα G-A*-*G* haplotype was associated with an increased risk of hand OA (1.61, 1.10-2.37, *p* = 0.01). Interactions were observed between *TNFα “-1031”* and *IL4R* Ser503Pro, *TNFα “-1031”* and *IL10 “-1082”*, and *TNFα “-863*” and *IL10 “-1082”* SNPs with regard to hand OA (*p* = 0.012, *p* = 0.0068, and *p* = 0.02, respectively). The carriage of the *TNFα “-1031”* minor allele doubled the risk (2.01, 1.26 - 3.22) only in women with the *IL4R* Ser/Ser genotype. Similarly, the *TNFα “-1031”* and *“-863*” minor alleles were associated with an increased risk of hand OA only in *IL10* G/G or A/A homozygotes (2.54, 1.45-4.47 and 2.60, 1.46-4.62, respectively) but not in heterozygotes (G/A).

**Conclusions:**

Our results suggest that the *TNF* α gene variants play a role in the etiology of hand OA. In addition, the findings are suggestive of a gene-gene interaction of the *TNF* α with *IL4R* and *IL10* genes.

**Electronic supplementary material:**

The online version of this article (doi:10.1186/1471-2474-15-311) contains supplementary material, which is available to authorized users.

## Background

Osteoarthritis (OA) is the most common joint disorder worldwide and rapidly increasing with ageing populations. OA is a dynamic process involving all the structures within the joint, *i.e.*, cartilage, synovial membrane and subchondral bone. It shows clinical heterogeneity in joint numbers and regions involved. Some patients may have only one site affected (hip, knee, or hand) affected (local OA), while others have clustered joint regions affected in a characteristic distribution (generalized OA) [1].

The hand is among the most frequently affected site in OA [2]. The prevalence of hand OA is higher in women than in men over the age of 50 [3]. Although the pathogenesis of hand OA is largely unknown, familial aggregation and heritability studies indicate a significant genetic role in addition to the involvement of mechanical (repetitive joint loading) and lifestyle related factors (*e.g.* obesity) [3, 4].

The development and progression of OA are nowadays believed to involve inflammation [5–8]. Chondrocytes, as well as synovial cells, of OA patients produce increased levels of pro-inflammatory cytokines, which affect metabolism and enhance the catabolism of all joint tissues affected in OA [5]. Among pro-inflammatory cytokines, interleukin-1β (IL1β) and tumor necrosis factor alpha (TNF*α*) seem prominent and of major importance to cartilage destruction as they are synthesized during the OA process [9–11]. *In vivo* studies have shown that these cytokines can act independently or in concert with other cytokines (e.g. IL6) in the induction and propagation of inflammation [12]. Synthesis of the IL1β and TNFα is inhibited by anti-inflammatory cytokines such as IL4, IL10 and IL13 [13]. On the other hand, expression of cytokine genes like *IL1* and *TNFα* is up-regulated in OA [14].

The gene encoding TNFα is located in the class III region of the major histocompatibility complex (MHC) which is the most gene-dense and polymorphic region of the entire genome [15]. TNFα is driving the inflammatory cascade [5]. IL4 in turn is a cytokine produced by T cells, which plays a major role in immunoglobulin E (IgE) production. Its signals are conferred to effector cells through binding to the alpha chain of IL4 receptor (IL4R). IL4 and IL4R are expressed by human articular chondrocytes; data suggest that mechanical stimulation induces the release of IL4 by human chondrocytes after the recognition and transduction of the mechanical signal by integrin [16, 17]. Therefore, the IL4R is an active autocrine or paracrine signaling molecule in a regulatory pathway in the maintenance of human articular cartilage structure and function [16]. Regulation of the structure and function of human articular cartilage occurs by mediating other biochemical responses to mechanical strain, proteoglycan synthesis, or altering the expressions of other extra cellular matrix (ECM) proteins involved in the pathogenesis of OA [16, 17].

So far, the evidence for involvement of genes propagating or inhibiting inflammation in the development or progression of OA is inconsistent, and the observed associations were not replicated in an independent population [18]. Most of the previous studies examined the role of a single gene in OA without taking into consideration the interaction of the genes participating in the regulation of balance between pro- and anti-inflammatory processes. Our group has reported the associations of the *IL1* extended haplotype and common *IL6* promoter variants with symmetrical DIP OA [19, 20].

The aim of the current study was to investigate the associations of common variants in the *TNFα* gene and their interactions with variants in the *IL4R* and *IL10* genes in relation to hand OA among middle-aged Finnish women, representing two occupations: dentists and teachers. The possible interactions of the *TNFα* with variants in the *IL1β* and *IL6* in influencing hand OA were also explored.

## Methods

### Study design and participants’ selection

This was a cross-sectional study, samples of which were taken randomly from two occupational groups. The study participants were identified from the registers of the Finnish Dental Association and the Finnish Teachers Trade Union. Four hundred and thirty-six women aged 45 to 63 were randomly selected from both occupational groups (altogether 872 subjects) by using the place of residence (Helsinki or its neighboring cities) as an inclusion criterion. Of those subjects who received the questionnaires in 2002, 542 (62% of the invited) participated in a clinical examination between October 2002 and March 2003. Of these, 294 (67% of the invited) were dentists and 248 (57% of the invited) teachers. Participation in the study was voluntary and based on informed consent. The study was approved by the Hospital District of Helsinki and Uusimaa Ethics Committee for Research in Occupational Health and Safety.

### Hand radiography and image analysis

Both hands of the study participants were radiographed by exposing Kodak X-ray films with Siemens X-ray equipment (48 kV, 10 mA, focus film distance = 115 cm; Siemens, Munich, Germany). The analogue radiographs were evaluated by an experienced radiologist who was blinded to the occupation, age, and all health data of the participants. Each distal interphalangeal (DIP), proximal interphalangeal (PIP), and thumb interphalangeal (IP) joint of both hands was graded separately, and classified for the presence of OA using a modified Kellgren and Lawrence (K-L) system [21]; the classification criteria were: grade 0 = no OA, grade 1 = doubtful OA, grade 2 = mild OA, grade 3 = moderate OA, and grade 4 = severe OA. The description of reference images used in the classification is given elsewhere [22]. The reliability of the readings was estimated by measuring intra-observer and inter-observer agreements (intraclass correlation) within a limited sample of radiographs and a second participating radiologist. The inter-observer agreement for OA ranged from 0.67 to 0.85 for DIP joints and from 0.39 to 0.61 for PIP joints. The intra-observer agreement for OA ranged from 0.73 to 0.88 for DIP joints and from 0.67 to 0.92 for PIP joints [22].

Participants who had at least three finger joints with radiographic OA of grade 2 to 4 were classified as having hand OA. Otherwise, the participants were classified as not having hand OA.

### Covariates

Weight was measured without shoes to the accuracy of 0.1 kg. Body mass index [BMI = weight (kg)/height (m)^2^] was calculated based on weight and self-reported height. BMI data was missing from one participant. Age, occupation, and BMI were considered as possible confounders in the analyses. The variants in the *IL1β* and *IL6* that was previously shown to influence hand OA were also included among the covariates.

### Genotyping analysis

Blood samples were taken from each study participant in the clinical examination and stored at +4°C until DNA extraction using a DNA extraction kit (PUREGENE ®DNA Purification Kit; Gentra Systems, Plymouth, MN, USA).

The *TNFα “-1031”* (rs1799964) and the *“-857”* (rs1799724) genotypes were determined by the TaqMan® SNP Genotyping Assay (Applied Biosystems, C___7514871_10 and C__11918223_10, respectively).

The *TNFα “-863*” (rs1800630) genotype was determined and the *“-857”* genotype re-determined by the Pyrosequencing® PSQ 96MA SNP/SQA system with PyroMark Assay Design self-designed protocol.

The *TNFα “-308”* (rs1800629) genotype was determined by PCR-RFLP method with *Nco* I (New England BioLabs (NEB) 10 U/μL) restriction enzyme. The primers were from Ozen *et al.*[23].

In the *TNFα “-1031”* locus the T-allele was denoted as the wild type allele and the C-allele as the variant allele, in the *“-863*” and *“-857”* loci the C-alleles were denoted as the wild type alleles and the A- and T-allele as the variant alleles, respectively, and in the *“-308”* locus the G-allele was denoted as the wild type allele and the A-allele as the variant allele.

The *IL4R* Ser503Pro (1507 T > C, rs1805015) and Ser752Ala (2254 T > G, rs1805016) polymorphisms were genotyped by PCR-based TaqMan® SNP Genotyping Assays (Applied Biosystems, C_234284_1 and C_8903091_10 respectively).

The *IL10 “-1082”* (rs1800896) SNP was genotyped with primers from Koch *et al*. [24].

An additional file has detailed description about the genotyping (see Additional file 1).

For quality control 10% of the genotyped samples were blindly repeated with 100% concordant results. Genotype data was available from all participants.

The earlier published *IL1β* “3954” (rs1143634), and *IL6 “174”* (rs1800795) genotyping has been described elsewhere [19, 20].

### Statistical analysis

The potential deviation of the allele frequencies from the Hardy-Weinberg equilibrium (HWE) was tested from controls using the chi-square test. The degree of pairwise linkage disequilibrium (LD) for four *TNFα* SNPs and two *IL4R* SNPs were calculated using SNPStats software [25]. Haplotypes were constructed and analyzed by the same software.

Logistic regression analysis was used to test the associations between SNPs and hand OA. For each SNP, a log-additive model of inheritance was fitted. To evaluate whether the observed association between the *TNFα* and OA was modified by variants in other cytokine genes, gene-gene interactions were tested for all *TNFα* SNPs by stratified logistic regression analyses.

Both crude and adjusted odds ratios (ORs) and their 95% confidence intervals (CIs) were calculated. The ORs were adjusted for the potential confounding factors, *i.e.*, age (continuous), occupation (dentists vs. teachers), and BMI (continuous). Since the crude and adjusted ORs did not differ significantly, only the adjusted ORs are shown in the results. In addition, the OR’s were further adjusted for genetic variants in the *IL1β* and *IL6*.

In addition to exploring whether the effect of the *TNFα* SNPs on hand OA were independent of the genetic variants in the *IL1β*[19] and *IL6*[20]*,* we estimated the individual and joint effects of the *TNFα* (*“-863*”), *IL1β* and *IL6* polymorphisms using the combinations of two dummy (0, 1) variables. First, we calculated the sum of the minor alleles of *IL1β* and *IL6*, by summing up the number of minor alleles of two SNPs. This was dichotomized (first dummy variable): 0 = non-carriers of any minor allele of the *IL1β* and *IL6* and 1 = carriers of at least one minor allele. For the *TNFα “-863*” SNP we used the dominant model, with the homozygous genotype of the major allele as the reference (second dummy variable).

All analyses were hypothesis driven. The statistical significance of the p-value was defined as the 1% level. P-values were adjusted for multiple testing using Sidák’s method [26]. We used SNPStats software [25] and SPSS 20.0 for the analyses.

## Results

The prevalence of hand OA with at least three affected finger joints was 29.5%, being higher among teachers (35.5%) than dentists (24.5%) (Table 1). Participants with hand OA were significantly older and had higher BMI than those without OA.

**Table 1:** **Description of the samples of female dentists and teachers aged 45–63, living in the metropolitan area of Helsinki, Finland**

	All	Dentists	Teachers
n (%)	542 (100)	294 (54)	248 (46)
Mean (SD) age (years)	54.0 (5.3)	53.7 (5.9)	54.3 (4.4)
Mean (SD) BMI (kg/m^2^)	24.5 (3.6)	23.9 (3.2)	25.1 (3.9)
Hand OA cases (%)	160 (29.5)	72 (24.5)	88 (35.5)

The genotype frequencies were in HWE in all of the studied polymorphic loci (Table 2). When adjusted for age, occupation and BMI, two *TNFα* SNPs (*“-1031”* and *“-863*”) were associated with hand OA (OR = 1.45, 95% CI 1.01-2.07, *p* = 0.04 and 1.55, 1.06-2.25, *p* = 0.02, respectively) (Table 3). Further adjustment for the *IL1β* and *IL6* SNPs had a negligible effect on the observed point estimates, though improving the estimate’s precision (*p* = 0.03 and *p* = 0.01, respectively). No statistically significant associations were found between the other two *TNFα* SNPs and hand OA. Neither were there associations between the SNPs in the *IL4R* or *IL10* and hand OA.

**Table 2:** **Description of studied SNPs**

Genes	Localization	SNP ID	Chro.	Position	MAF										HWE
					Total (n = 1084)		OA Cases (n = 320)		OA Controls (n = 764)		1000 Genomes Finland# (n = 186)		HapMap¤ (n = 226)		p-value in controls
					%	n	%	n	%	n	%	n	%	n	
*TNFα*	*−1031*	rs1799964	6	31542308	18	191	21	66	16	125	19	35	21	48	0.41
*TNFα*	*−863*	rs1800630	6	31542476	16	168	19	60	14	108	15	27	15	34	0.79
*TNFα*	*−857*	rs1799724	6	31542482	6.4	69	5.6	18	6.7	51	6.5	12	NA/5.9*	10	0.81
*TNFα*	*−308*	rs1800629	6	31543031	13	144	15	47	13	97	13	24	17	39	0.59
*IL4R*	*Ser752Ala*	rs1805016	16	27374927	3.2	35	4.1	13	2.9	22	5.4	10	6.3	14	0.56
*IL4R*	*Ser503Pro*	rs1805015	16	27374180	13	139	14	44	12	95	15	27	15	34	0.17
*IL10*	*−1082*	rs1800896	1	206946897	42	453	42	133	42	320	39	73	47	120	0.84
*IL1β*	*3954*	rs1143634	2	113590390	27	294	31	98	26	196	24	44	21	47	0.97
*IL6*	*174*	rs1800795	7	22766645	44	475	48	153	42	322	44	82	47	105	0.88

#1000 genomes European sub-population, Finnish in Finland.

¤HapMap population is CEU: Utah residents with Northern and Western European ancestry from the CEPH collection.

*1000 genomes, population is CEU: Utah residents (CEPH) with Northern and Western European ancestry, n = 170.

*SNP* single nucleotide polymorphism, *MAF* minor allele frequency, *HWE* Hardy-Weinberg equilibrium, *OA* osteoarthritis.

**Table 3:** **Association of the variants in the cytokine genes with hand OA**

			Hand OA n = 160 (542)			
Genes	Localization	SNP ID	OR (95% CI)^1^	p-value	OR (95% CI)^2^	p-value
*TNFα*	*−1031*	rs1799964	1.45 (1.01-2.07)	0.04	1.47 (1.02-2.13)	0.03
*TNFα*	*−863*	rs1800630	1.55 (1.06-2.25)	0.02	1.61 (1.10-2.36)	0.01
*TNFα*	*−857*	rs1799724	0.77 (0.44-1.35)	0.35	0.77 (0.44-1.35)	0.36
*TNFα*	*−308*	rs1800629	1.25 (0.83-1.86)	0.29	1.19 (0.79-1.79)	0.39
*IL4R*	*Ser752Ala*	rs1805016	1.41 (0.67-2.99)	0.37	1.47 (0.69-2.32)	0.33
*IL4R*	*Ser503Pro*	rs1805015	1.17 (0.77-1.77)	0.47	1.17 (0.76-1.78)	0.48
*IL10*	*−1082*	rs1800896	0.96 (0.73-1.27)	0.80	0.95 (0.71-1.25)	0.70
*IL1β*	*3954*	rs1143634	1.39 (1.02-1.88)	0.03		
*IL6*	*174*	rs1800795	1.21 (0.92-1.59)	0.18		

^1^ORs and their 95% CIs were adjusted for age, occupation and BMI.

^2^ORs and their 95% CIs were adjusted for age, occupation, BMI and carriage of the minor allele of the *IL1β* (rs1143634) or/and *IL6* (rs1800795) SNPs.

*OA* osteoarthritis, *SNP* single nucleotide polymorphism, *OR* odds ratio, *CI* confidence interval, *BMI* body mass index.

Statistically significant interactions were found between the *TNFα “-1031”* and *IL4R* Ser503Pro SNPs, *TNFα “-1031”* and *IL10 “-1082”* SNPs, and *TNFα “-863*” and *IL10 “-1082”* SNPs and hand OA (*p* = 0.012, *p* = 0.0068, and *p* = 0.02, respectively). The carriage of the *TNFα* (*“-1031”*) minor allele was associated with a double risk of hand OA (2.01, 1.26 - 3.22) in women with the *IL4R* Ser/Ser genotype (Table 4). Similarly, the *TNFα “-1031”* and *“-863*” minor alleles were associated with an increased risk of hand OA only in *IL10* G/G or A/A homozygotes (2.54, 1.45-4.47 and 2.60, 1.46-4.62, respectively) but not in heterozygotes (G/A).

**Table 4:** **Interaction of the** ***TNFα*** **SNPs with the** ***IL4R*** **and** ***IL10*** **SNPs in their effect on hand OA**

		n	OR	95% CI	p-value
***IL4R***	**TNFα** *“-1031”*				0.01
Ser/Ser	T/T	67/270	1.00		
	T/C – C/C	52/138	2.01	1.26-3.22	
Ser/Pro-Pro/Pro	T/T	33/94	1.00		
	T/C – C/C	8/39	0.55	0.22-1.40	
***IL4R***	**TNFα** *“-863*”				0.05
Ser/Ser	C/C	71/285	1.00		
	C/A – A/A	33/99	2.09	1.29-3.27	
Ser/Pro-Pro/Pro	C/C	48/123	1.00		
	C/A – A/A	8/34	0.72	0.28-1.87	
***IL10***	**TNFα** *“-1031”*				0.007
G/A	T/T	55/175	1.00		
	T/C – C/C	20/83	0.80	0.43-1.51	
A/A-G/G	T/T	29/120	1.00		
	T/C – C/C	27/66	2.54	1.45-4.47	
***IL10***	**TNFα** *“-863*”				0.02
G/A	C/C	56/186	1.00		
	C/A – A/A	19/72	0.94	0.49-1.80	
A/A-G/G	C/C	48/198	1.00		
	C/A – A/A	37/85	2.60	1.46-4.62	

Odds ratios (ORs) and 95% confidence intervals (CIs) are adjusted for age, occupation and body mass index (BMI).

*SNP* single nucleotide polymorphism, *OA* osteoarthritis, *OR* odds ratio, *CI* confidence interval.

We also examined the individual and joint effects of the *TNFα “-863*”, *IL1β,* and *IL6* polymorphisms on hand OA. The risk of hand OA was the highest in the carriers of the minor alleles in all three genes (4.37, 1.84-10.38, *p* = 0.001). Somewhat lower risks were observed for carriers of the *TNFα “-863*” minor allele (3.73, 1.28-10.85, *p* = 0.016) and carriers of minor alleles of *IL1β* and *IL6* SNPs (2.89, 1.29-6.48, *p* = 0.010).

The degree of pairwise LD between three *TNFα* SNPs (*“-1031”*, *“-863*”, and *“-308”*) was high; the *“-1031”* and *“-863*” SNPs were in complete linkage (D’ = 1, r^2^ = 0.93, *p* < 0.0001), and the *IL4R* Ser503Pro and Ser752Ala polymorphisms were also in a strong LD (D’ = 0.998, r^2^ = 0.48, *p* < 0.0001). The three *TNFα* promoter polymorphisms composed a total of four haplotypes. The most common of these haplotypes was T-C-G (0.69), followed by G-A-G (0.16), T-C-A (0.13), and G-C-G (0.02). The two *IL4R* polymorphisms, on the other hand, composed three haplotypes, *i.e.*, Ser-Ser (0.87), Ser-Pro (0.10) and Ala-Pro (0.03).

The *TNFα G-A*-*G* haplotype was associated with an increased risk of hand OA when adjusted for age, occupation, and BMI (1.61, 1.10-2.37, *p* = 0.01) (Table 5). There was no difference between participants with and without hand OA in the *IL4R* haplotype distribution.

**Table 5:** **Association of haplotypes with hand OA**

	OA- (n = 382)	OA + (n = 160)	OR (95% CI)	p-value
***TNFα-1031-863 -308***				0.07*
T-C-G	0.71	0.65	1.00	
C-A-G	0.14	0.19	1.61 (1.10-2.37)	0.01
T-C-A	0.13	0.15	1.35 (0.89-2.03)	0.16
C-C-G	0.02	0.02	0.93 (0.33-2.58)	0.88
***IL4R***				0.65*
Ser-Ser	0.88	0.86	1.00	
Ser-Pro	0.10	0.10	1.08 (0.67-1.74)	0.73
Ala-Pro	0.02	0.04	1.43 (0.67-3.03)	0.37

ORs and their 95% CIs were adjusted for age, occupation and BMI.

*Global haplotype association p-value.

## Discussion

We investigated whether the *TNF* α promoter polymorphisms are associated with hand OA among Finnish women. The minor alleles of the *TNFα* “*-1031”* and “*-863”* loci, as well as their haplotype, were found to be associated with an increased risk of hand OA. The observed associations were independent of the variants in the *IL1β* and *IL6* genes. Furthermore, our findings suggest that the effect of *TNFα* polymorphisms on hand OA is modified by the variants within the *IL4R* and *IL10* genes.

A traditional paradigm of OA as a “wear and tear” disease leading to the loss of cartilage has been revised. Nowadays, OA is considered a complex disease with inflammatory mediators released by cartilage, bone and synovium [8]. TNFα is one of the most typical pro-inflammatory cytokines that along with IL1β is connected with cartilage destruction. These two cytokines, which are produced by chondrocytes, mononuclear cells, osteoblasts and synovial tissues, induce the production of a number of inflammatory and catabolic factors [5]. Among the four polymorphic loci studied here, only the *TNFα “-308”* locus has been shown to affect the TNFα protein levels, the minor allele of the SNP was associated with increased TNFα production in response to various stimuli [27, 28]. However, also opposite observations, *e.g.*, no effect on the protein levels or lowered protein levels, have been reported [29, 30]. When studying the above SNPs with F-SNP-program that is freely available on the internet (http://compbio.cs.queensu.ca/F-SNP/) connected to the main databases, and computationally predicting functional SNPs, all four SNPs are predicted to be functional as they seem to be in the transcription factor binding site [31]. However, the protein level alteration by the studied SNPs still remains unsolved and needs to be further studied.

The few studies that examined associations between *TNFα* polymorphisms and knee or hip OA have given conflicting results [32]. To our knowledge, this is the first study to report an association between the *TNFα* variant alleles and hand OA. TNFα can act independently or in concert with other cytokines (*e.g*., IL1β, IL4, IL6, and Il10) to initiate and expand inflammation [5]. Ignorance of the complex interrelationships between pro- and anti-inflammatory cytokines might be the reason for failure to detect an association between the *TNFα* polymorphisms and OA. It has been suggested that the combined use of information from multiple markers may be more effective to reveal the association between a genomic region and a trait than a single marker analysis [33].

Previously our group reported the associations of the *IL1* and *IL6* gene polymorphisms with hand OA [19, 20]. The current findings suggest that *TNFα* promoter polymorphisms may increase the risk of hand OA independently of the polymorphisms in the *IL1* and *IL6* genes, and that the effect attributed to combination of variants in all three genes is larger, but less than additive.

When we examined the association of the *TNFα* polymorphisms with hand OA taking into consideration the variants of other cytokine genes, the *IL4R* and *IL10* polymorphisms appeared to act as effect modifiers.

Vargiolu *et al*. [34] reported an association of genetic variants in the coding region of the *IL4R* gene with hand OA among men and women in the age range of 41 to 84 years. However, we failed to replicate this association among our participants. Differences between the study populations, OA phenotypes, and minor allele frequencies might be the reasons for discrepancies in the findings. Our participants were younger (mean age 53 years) than in the study by Vargiolu and coworkers, which may partly explain the difference of the prevalence of hand OA between their and our study (55.6% and 29.5%, respectively).

Naturally occurring anti-inflammatory cytokines such as IL10 inhibit the synthesis of IL1 and TNFα [13]. The *IL10 “-1082”* polymorphism has been shown to affect the level of the protein production: the A-allele is connected with a significantly higher protein production than the G-allele [35]. This polymorphism was also associated with rheumatoid arthritis in a meta-analysis [36]. As to its role in DIP OA, no association was found in the Dutch population [37]. Similarly, the *IL10 “-1082”* did not associate with hand OA in our study.

A major strength of the study was that all study participants were of the ethnically relatively homogenous Finnish origin. Each ethnic group has its own set of environmental and genetic factors that contribute to the disease risk, and differences in allelic frequency often affect our ability to detect a susceptibility allele. The Finnish population is known to be a genetic isolate, which originated from a small founder population some 2000 years ago. Therefore, the Finnish population with the relatively homogenous gene pool [38] offers an optimal material for association studies.

Another strength of our study is that we analyzed haplotypes in addition to SNPs. Grouping of SNPs in haplotypes generally leads to a stronger association with the phenotype than individual polymorphisms.

Further, the prevalence of hand OA was similar to that seen in other studies [39–41], and major potential confounders were controlled for in the statistical analyses.

A limitation of our study is the relatively small number of participants, leading to reduced power to detect small effects and an increased likelihood of spurious findings. This needs to be considered while interpreting the observed associations. Another obvious limitation is the fact that our study participants were all women and consequently the results cannot be generalized to men.

## Conclusions

Our results suggest that variants in the *TNF* α gene play a role in the etiology of hand OA in Finnish women. In addition, the findings are suggestive of a gene-gene interaction of the *TNF* α with the *IL4R* and *IL10* genes. However, these findings should be considered with caution until replicated in other study population.

## Electronic supplementary material

Additional file 1:Detailed protocols for genotyping.(DOC 34 KB)

## Abbreviations

TNFα: Tumor necrosis factor alpha
IL6: Interleukine 6
IL1: Interleukine 1
OA: Osteoarthritis
BMI: Body mass index
PCR: Polymerase chain reaction
SNP: Single nucleotide polymorphism
DIP: Distal interphalangeal
PIP: Proximal interphalangeal
IP: Interphalangeal
IL1B: Interleukin 1, beta
K-L: Kellgren and Lawrence
RFLP: Restriction fragment length polymorphism
HWE: Hardy-Weinberg equilibrium
LD: Linkage disequilibrium
OR: Odds ratio
CI: Confidence interval
MHC: Major histocompatibility complex.
