# Supplementary material for: Variations in the TNFα gene and their interactions with the IL4R and IL10 genes in relation to hand osteoarthritis
Source: BMC Musculoskelet Disord. 2014 Sep 24;15:311. doi: 10.1186/1471-2474-15-311 (PMC4181701; doi:10.1186/1471-2474-15-311)
Supplement: Supplementary file 1 — Additional file 1:Detailed protocols for genotyping.(DOC 34 KB) [file 12891_2014_2249_MOESM1_ESM.doc]

Additional file 1.

Detailed protocols for genotyping:

The *TNFα "-863*" (rs1800630) genotype was determined and the *"-857"* genotype re-determined by the Pyrosequencing**®** PSQ 96MA SNP/SQA system with PyroMark Assay Design self-designed protocol. The PCR primers were: Forward 5'-GGT AGG AGA ATG TCC AGG GCT ATG-3' and Reverse 5'-Biotin-ACT CCC TGG GGC CCT CTA-3'. The 50 µl PCR reaction mixture was composed of 5 µl 10x Buffer, 4 µl dNTP (2.5mM), 3 µl MgCl_2_ (1.5mM), 0.1 µl Forward Primer (100pmol/µl), 0.1 µl Reverse Primer (100pmol/µl), 39.65 µl mQ H_2_O, 0.15 µl Taq polymerase (5U/µl), and 1 µl DNA (10 ng/µl). The PCR protocol was as follows: initial denaturation of 5 s at 95°C followed by 35 cycles of 15 s at 95°C, 30 s at 56°C, and 15 s at 72°C, and final elongation of 5 min at 72°C. The PCR product was prepared for cleaning with PyroMark Binding buffer and Streptavidin Sepharose High Performance (GE Healthcare) beads. The templates were cleaned and denatured to single strand form by treatment with 70 % Ethanol (Aa), PyroMark Denaturation Buffer, PyroMark Washing Buffer, and mQ water in Pyrosequencing Washing Station. The Sequencing Primer 5'-TCG AGT ATG GGG ACC-3' was attached to the template with PyroMark Annealing Buffer for 2 min in 80°C. The Pyrosequencing run was made in the dispensation order of: G C A C G T A C T C G A G.

The *TNFα”-308”* (rs1800629) genotype was determined by PCR-RFLP method with *Nco*I (New England BioLabs (NEB) 10U/µL) restriction enzyme. The primers was from Ozen *et al.* . Our 30 µl PCR reaction mixture was composed of 3 µl 10x Taq-buffer, 2.4 µl dNTP (2.5mM), 0.9 µl MgCl_2_, 0.15 µl Forward Primer (100pmol/µl), 0.15 µl Reverse Primer (100pmol/µl), 18.2 µl mQ H_2_O, 0.2 µl Taq polymerase (5U/µl), and 5 µl DNA (10 ng/µl). The PCR protocol was as follows: initial denaturation of 2 min at 94°C followed by 35 cycles of 30 s at 94°C, 30 s at 60°C, and 45 s at 72°C, and final elongation of 5 min at 72°C. Digestion reaction mix included 1.5 µl NEBuffer 4, 0.3 µl Nco I (NEB 10U/µL), 8.2 µl mQ H_2_O and 5 µl PCR product. Digestion was done in +37°C for 3 hours. The restriction fragments were separated by electrophoresis in 3 % agarose gel with ethidium bromide.

In the *TNFα "-1031"* locus the T-allele was denoted as the wild type allele and the C-allele as the variant allele, in the *"-863*" and *"-857"* loci the C-alleles were denoted as the wild type alleles and the A- and T-allele as the variant alleles, respectively, and in the *"-308"* locus the G-allele was denoted as the wild type allele and the A-allele as the variant allele.

The *IL4R* Ser503Pro (1507T>C, rs1805015) and Ser752Ala (2254T>G, rs1805016) polymorphisms were genotyped by PCR-based TaqMan® SNP Genotyping Assays (Applied Biosystems, C_234284_1 and C_8903091_10 respectively).

The *IL10 “-1082”* (rs1800896) SNP was genotyped with primers from Koch *et al*. (2001) . The 30 µl PCR reaction mixture was composed of 3 µl 10x Taq-buffer, 2.4 µl dNTP (2.5mM), 0.9 µl MgCl_2_, 0.15 µl Forward Primer (100pmol/µl), 0.15 µl Reverse Primer (100pmol/µl), 18.2 µl mQ H_2_O, 0.2 µl Taq polymerase (5U/µl), and 5 µl DNA (10 ng/µl). The PCR protocol was as follows: initial denaturation of 2 min at 94°C followed by 35 cycles of 30 s at 94°C, 30 s at 55°C, and 45 s at 72°C, and final elongation of 5 min at 72°C. Digestion reaction mix included 1.5 µl Buffer R+, 0.3 µl Xag I (Fermentas 10U/µl), 8.2 µl mQ H_2_O and 5 µl PCR product. Digestion was done in +37°C for 3 hours. The restriction fragments were separated by electrophoresis in 3 % agarose gel with ethidium bromide.
